# Case of Patient with AML with Complex Karyotype including Ultra-Rare t(4;8)(q32;q13), t(4;11)(q21;p15) and Familial Aggregation of Myeloid Malignancies

**DOI:** 10.3390/medicina58010105

**Published:** 2022-01-10

**Authors:** Sławomir Milczarek, Ewa Studniak, Bartłomiej Baumert, Michał Janowski, Wioleta Bonda, Joanna Pietrzak, Aleksandra Łanocha, Edyta Paczkowska, Barbara Zdziarska, Bogusław Machaliński

**Affiliations:** 1 Department of General Pathology, Pomeranian Medical University, 70-111 Szczecin, Poland; slawek.milczarek@gmail.com (S.M.); bbaumert@pum.edu.pl (B.B.); edyta.paczkowska@pum.edu.pl (E.P.); 2 Department of Hematology and Transplantology, Pomeranian Medical University, 71-252 Szczecin, Poland; ewa.studniak@gmail.com (E.S.); janowskimm@gmail.com (M.J.); aleksandra.lanocha@pum.edu.pl (A.Ł.); barbara.zdziarska@pum.edu.pl (B.Z.); 3 Cytogenetic Unit, Independent Public Clinical Hospital nr 2, Pomeranian Medical University, 70-111 Szczecin, Poland; wioleta.bonda@gmail.com (W.B.); joanna311@wp.pl (J.P.)

**Keywords:** AML, t(4;8)(q32;q13), t(4;11)(q21;q15), familial aggregation, MPN

## Abstract

We present a unique case of a young woman with acute myeloid leukemia (AML) with complex karyotype. The presence of the t(4;11)(q23;p15) is extremely rare in myeloid leukemias, while t(4;8)(q32;q13) has not yet been described in any leukemia reference. Another interesting issue is the familial aggregation of myeloid malignancies and worse course of the disease in each subsequent generation, as well as an earlier onset of the disease. Our report emphasizes the need for thorough pedigree examination upon myeloid malignancy diagnosis as there are relatives for whom counseling, gene testing, and surveillance may be highly advisable.

## 1. Introduction

Inherited forms of myeloid malignancies are believed to be rare, although the prevalence and exact incidence remain unknown. Myeloid neoplasms with germ line predisposition were recognized as a separate disease entity in the World Health Organization (WHO) classification of hematological cancers and by the European LeukemiaNet (ELN) in 2016 [1,2], which increased awareness of these forms of the disease. There are large population studies investigating the risk of developing hematological malignancies among relatives of hematooncological patients. A large Swedish cohort showed significantly increased risk of all myeloid malignancies including acute myeloid leukemia (AML), polycythemia vera (PV), myelodysplastic syndrome (MDS), and essential thrombocythemia (ET) among relatives [3]. Interestingly, the correlation was stronger for younger patients [3]. Increased familial clustering of myeloproliferative neoplasms (MPNs) was previously confirmed [4]. A subset of MPN patients transform to AML, defined as blast phase (MPN-BP). The molecular mechanisms underlying blast transformation have not been fully elucidated, and the specific genetic and epigenetic events governing leukemogenesis remain unclear [5]. In contrast, a diagnosis of chronic myeloid leukemia (CML) does not appear to have an impact on the development of CML, AML, or MPN in relatives [6].

Herein, we report a history of family aggregation of MPNs with a worse course of the disease in each subsequent generation and earlier onset of the disease. The family history goes on and many factors indicate an increased risk of developing hematological cancers in subsequent family members.

## 2. Case Report

A 34-year-old female patient with a history of asthma and inflammatory bowel disease was admitted to the Department of Hematology and Transplantology, Pomeranian Medical University in Szczecin, with suspected AML. The main complaints included general malaise, anorexia, palpitations, and tinnitus, which had been increasing for about two months. On admission, the patient was in good clinical condition, with skin pallor as the sole abnormality on physical examination. Initial laboratory tests revealed severe macrocytic anemia with hemoglobin level 5.6 g/dL, leukopenia with absolute neutrophil count (ANC) 0.24 G/L, and lactate dehydrogenase elevation. Plain infection, B12 vitamin, folic acid deficiency, and viral infections (hepatitis B and C and human immunodeficiency virus) were excluded. Conventional chest X-ray and abdominal ultrasonography were unremarkable. The peripheral blood smear showed about 60% myeloblasts. Bone marrow (BM) aspirate revealed an infiltration of myeloblasts with Auer rods exceeding 85% of the cells. The cytometric phenotyping showed about 90% of precursor cells with the following phenotype: CD13, CD33, CD34, CD117, CD38, CD31, CD97, CD123, which confirmed the diagnosis of AML M0/M1 according to the former FAB classification. Based on molecular tests, the presence of the *FLT3-ITD*, *NPM1*, and *CEBPA* mutations was excluded.

**Figure 1:**
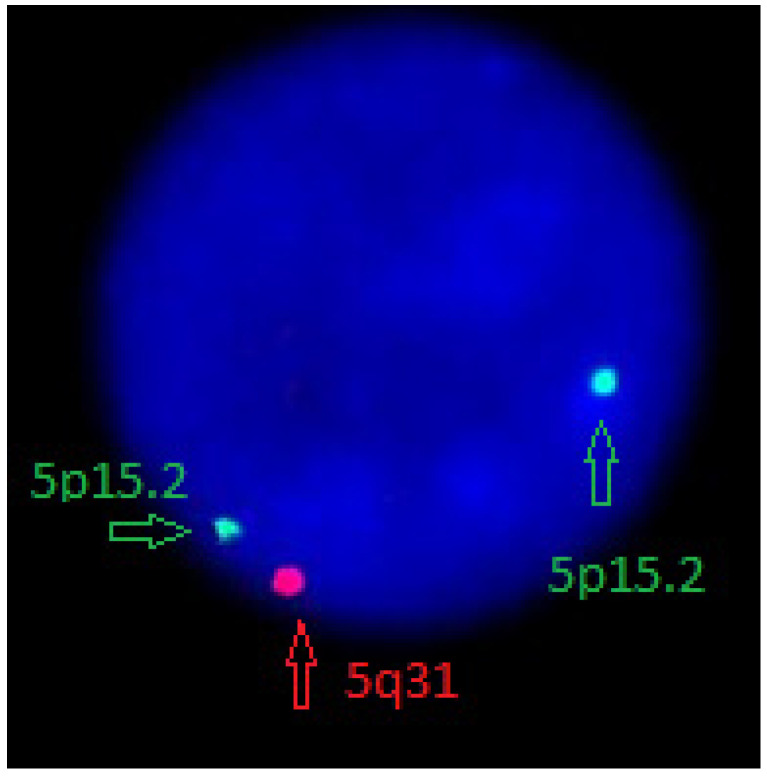
FISH probe: LSI EGR1/D5S23, D5S721 Dual Color Probe Kit (CE). Abnormal cell containing the deletion: the one orange and two green (1O2G) signal pattern is observed.

Metaphase cells from short-term 24 h unstimulated cultures were examined. Bone marrow cytogenetic testing exhibited complex karyotype described according to the International System of Human Cytogenomic Nomenclature (ISCN) 2020 as 46,XX,t(4;8)(q32;q13),t(4;11)(q23;p15),del(5)(q15q34)[15]/47,XX,sl,+der(4)t(4;8)(q?32;q?13). FISH test has confirmed interstitial deletion of the long arm of chromosome 5: nuc ish(D5S23,D5S721x2),(EGR1x1)[180/200], (D5S23,D5S721x2),(CSF1Rx1)[180/200] (Figure 1).

Translocation between the long arm of chromosome 4 in the q32 region and the long arm of chromosome 8 in the q13 region was observed in all analyzed metaphases. In order to verify the origin of this aberration, cells from 72 h cultures stimulated with phytohemagglutinin (PHA) and constitutional karyotype from peripheral blood have been assessed and showed normal female 46,XX results. The study has been extended by the assessment of Array Comparative Genomic Hybridization (aCGH, 60k arrays) technique with the following result: arr [37hcg]4p16.3p11(45882_49083290)x3,4q11q32.1(52685197_156232623)x3,5q22.3-q35.3x1,8q11.1-q24.3x3.

MLPA technique has been used with the Hematologic Malignancies (MRC Holland P377-A3) for the detection of deletions or duplications in the 2p (*MYCN, ALK*), 5q (*MIR145, EBF1, MIR146A*), 6q, 7p12 (*IKZF1*), 7q, 8q24 (*MYC*), 9p (*MTAP, CDKN2A, CDKN2B, PAX5*), 10q23 (*PTEN*), 11q22 (*ATM*), 12p13 (*ETV6, CCND2, MDM2*), 12q, 13q14 (*RB1, MIR15A, DLEU2, DLEU1*), 17p13 (*TP53*), 17q, chr 18 and chr 19, and 21q22 (*RUNX1*). No aberration has been observed in blood cells except for deletion of 5q31.1.

**Figure 2:**
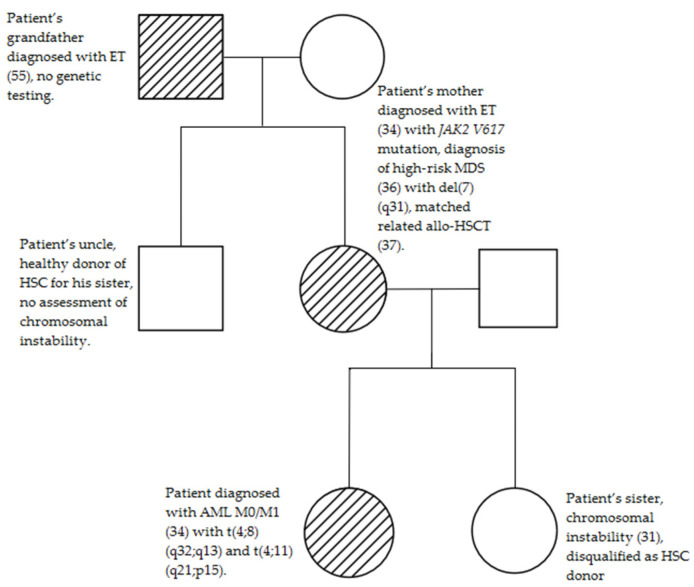
The pedigree of the patient’s family. The numbers in parentheses represent the age of onset of the disease.

Profound pedigree analysis has been performed, revealing myeloid neoplasm aggregation in previous generations (Figure 2).

The patient’s grandfather was diagnosed with ET in his fifties, but lack of written documentation prevents further inspection. The patient’s mother was diagnosed with ET at the age of 34 years with *JAK2 V617F* mutation. She was initially treated with hydroxyurea and subsequently with anagrelide. After one year of treatment, she was re-assessed due to progressive anemia. Her karyotype could not be assessed, but simultaneous FISH analysis showed a high risk of clonal deletion of the long arm of chromosome 7 in q31. Ultimately, the diagnosis of high-risk MDS has been established. At the age of 37, she underwent matched related allogeneic hematopoietic stem cell transplantation (allo-HSCT) with her brother being the donor. Since that time, she has remained in complete remission. There are no data regarding any hematological diseases affecting her brother to the present time.

**Figure 3:**
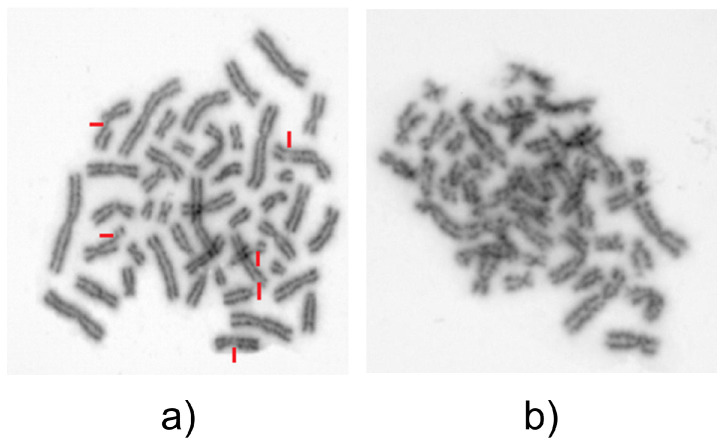
Bleomycin test: (**a**) 6 breaks/cell; (**b**) >50 breaks/cell.

Our patient was treated with standard induction therapy consisting of daunorubicin, cytarabine, and cladribine (DAC) with two subsequent high-dose cytarabine consolidation cycles. After achieving a complete remission, she underwent matched unrelated allo-HSCT preceded by myeloablative conditioning. After twelve months, she exhibited molecular relapse with progressively declining donor chimerism. Rapid immunosuppression taper and fourfold donor lymphocyte infusions (DLI) were administered. Despite the treatment, she was eventually diagnosed with full clinical relapse, phenotypically and genetically identical to initial AML. The induction treatment was resumed according to the previously used DAC protocol, followed by one consolidation cycle with high dose-cytarabine. She was eligible for another allo-HSCT, and haploidentical transplantation from the patient’s sister was considered. Having in mind the family history, additional genetic tests were performed and revealed bleomycin-induced chromosomal instability in her sister’s cells, excluding her from the donation. Bleomycin is already widely used in the evaluation of chromosomal instability in patients with clinical suspicion of Fanconi anemia [7] or ataxia–telangiectasia [8], but it is not used as a supplementary examination or routine before allo-HSCT. It is based on scoring of chromatid breaks on in vitro lymphocytes exposed to bleomycin during the late G2 phase of the cell cycle. Sister’s test resulted in an increased number of breaks/cell (1.27 vs. 0.00 for the control) and an increased number of damaged cells (26% vs. 0%) (Table 1, Figure 3). Regarding the result, increased chromosomal instability is diagnosed from 0.8 (number of breaks/cell) and high chromosomal instability from 1.0 [9,10].

Ultimately, she received a matched unrelated allograft. Our patient died during the second transplantation procedure due to infective complications. Interestingly, this woman in the distant past was a stem cell donor for matched unrelated allo-HSCT, and subsequently donor for the DLI. The long-term outcome of this procedure remains unknown.

Our patient’s motivation for being the bone marrow donor was the disease of her mother and the inconvenience of waiting for a suitable donor.

## 3. Discussion

Our report highlights some intriguing insights to debate. The (4;11)(q21;p15) translocation fuses the *NUP98* and *RAP1G* genes and is usually associated with T-cell acute lymphocytic leukemia [11]. However, the t(4;11)(q23;p15) seems to be extremely rare, and its exact incidence in myeloid leukemias is difficult to establish. To our knowledge, only a few cases have been described so far. In one case, a 36-year-old male was initially diagnosed with AML-M3. After failure of the initial treatment, the patient underwent allogeneic bone marrow transplantation (allo-BMT). Eleven months after BMT, the patient was diagnosed with recurrent AML, showing morphology consistent with AML-M4. Despite second allogeneic transplantation, the patient died one month after the procedure [12]. The second case concerned t(4;11)(q23;p15) and expression of the *NUP98::RAP1GDS1* fusion product detected in a 60-year-old woman with AML-M0 [13]. She received intensive chemotherapy with intention to proceed to transplant. As no allogeneic sibling donor was available for the patient, consolidation therapy consisted of busulfan and cyclophosphamide and auto-HSCT. After 2 months, she had a relapse, and after 8 months from initial diagnosis, the patient died as no curative therapy was available. The clinical course of patients with *NUP98* rearrangements seems aggressive and the outcome of treatment is disappointing. We hope that a better understanding of the disease process could help identify new treatment strategies to contend with this poor clinical outcome [14]. Large-scale screening should provide more *NUP98*-rearranged cases, allowing more accurate estimations of their incidence and proper assessment of the prognostic value of these rearrangements and deletions.

A particularly interesting observation is the presence of t(4;8)(q32;q13) in all karyotyped metaphases, which has not yet been described in the leukemia references. The additional der(4)t(4;8)(q?32;q?13) was most likely caused by clonal evolution and indicates disease progression.

Another interesting issue is the familial aggregation of myeloid malignancies and worse course of the disease in every subsequent generation of the family. Genetic molecular testing is the foundation of modern diagnostics. Most of the involved genes such as *CEBPRA, RUNX1*, *GATA2*, and *ETV6* are also well-known somatic driver mutations in de novo MDS/AML. It is critical to confirm the molecular findings with specialized germline testing on constitutional DNA. Skin fibroblast culture is considered the gold standard to obtain germline DNA. Unfortunately, the remaining family members refused to undergo additional testing and fibroblast analysis was not performed. Therefore, we could not exclude germline mutations. Sud et al. [3] described increased risk of all myeloid malignancies in first-degree relatives of patients with myeloid malignancies. The association between family history and increased risk was highest for patients with AML, whose relatives had MDS and PV.

## 4. Conclusions

Chromosomal instability creates cytogenetic diversity and is a common feature in hematological malignancies, especially in all types of acute myeloid leukemia (de novo AML, secondary AML, and therapy-related AML), increasing the risk of the disease [15]. Considering the abnormal result of the examination of the patient’s sister, we advised careful monitoring for hematological abnormalities and thorough examination if any alterations occur. Interestingly, our report emphasizes the need for thorough pedigree examination upon myeloid malignancy diagnosis as there are relatives for whom counseling, gene testing, and surveillance may be highly advisable. Parallelly, the fact that our patient was a stem cell donor suggests the need for change in the management of stem cell donation. We emphasize the need for genetic testing of potential donors as there are cases of donor-derived malignancies following transplantation [16].

## Figures and Tables

**Table 1:** Assessment of bleomycin-induced chromosomal instability.

	Test Sample without Bleomycin (*n* = 100 Cells)	Test Sample with Bleomycin (*n* = 100 Cells)	Control Sample without Bleomycin (*n* = 50 Cells)	Control Sample without Bleomycin (*n* = 50 Cells)
Number of chromatid breaks	0	127	0	0
Breaks/cell ratio	0	1.27	0	0
% of damaged cells	0	26	0	0

## Data Availability

The datasets used and/or analyzed during the current study are available from the corresponding author on reasonable request.
